# Effects of High-Pressure Processing and Lipid Fortification on Hoki (*Macruronus novaezelandiae*) Surimi Structure, Oxidative Stability, and In Vitro Digestion

**DOI:** 10.3390/foods15203658

**Published:** 2026-10-15

**Authors:** Mahmud Kobid, Rae Recy B. Bagonoc, Lovedeep Kaur, Michelle Ji Yeon Yoo

**Affiliations:** 1 School of Science, Auckland University of Technology, Auckland 1010, New Zealand; rvb9483@autuni.ac.nz (M.K.);; 2 School of Food Technology and Natural Sciences, Massey University, Palmerston North 4410, New Zealand

**Keywords:** high-pressure processing, hoki surimi, *Macruronus novaezelandiae*, lipid fortification, fish protein gels, water-binding capacity, lipid oxidation, free amino acid release, in vitro digestion, microstructure

## Abstract

High-pressure processing (HPP) and lipid fortification affect surimi functionality, but their combined effects on oxidation and digestion-related responses remain insufficiently characterised. Hoki (*Macruronus novaezelandiae*) surimi containing 10% (*v*/*w*) canola oil, flaxseed oil, or no-added-oil control was treated at 0–600 MPa. Technological properties, colour, thiobarbituric acid reactive substances (TBARS), microstructure, protein banding, and free amino acid (FAA) release during in vitro digestion were assessed. Oil fortification affected moisture and fat content, while pressure had limited effects on bulk proximate composition. Pressure reduced cooking loss and increased water-binding capacity (WBC) and slice shear force (gel resistance to shearing), with WBC showing an oil fortification × pressure interaction (*p* < 0.001). Oil-containing gels showed higher WBC, lightness, yellowness, and whiteness than the control. TBARS values were affected by oil fortification, pressure, and their interaction (*p* < 0.001), were highest in flaxseed samples, and decreased with pressure in the measured subset. Microstructure indicated improved network integration at moderate pressure, whereas higher pressure produced denser, less uniform structures. FAA release was governed mainly by digestion phase (*p* < 0.001), with limited, non-linear pressure effects. Overall, HPP and lipid fortification influenced hoki surimi through matrix-level changes in functionality, oxidation, structure, and FAA release.

## 1. Introduction

Surimi is a myofibrillar protein-based gel system in which processing conditions strongly influence water retention, texture, structural stability, and overall product quality. Gel formation is primarily governed by myosin, whose unfolding and subsequent intermolecular interactions support the development of a three-dimensional protein network. In surimi systems, salt-induced solubilisation of myofibrillar proteins is essential for effective gel formation, as it promotes myosin unfolding and subsequent polymerisation during setting and thermal processing. Exposure of hydrophobic regions and reactive sulfhydryl groups facilitates hydrophobic interactions, hydrogen bonding, and disulfide cross-linking, contributing to network development and functional properties such as texture and water retention [1,2].

The intermediate setting stage further contributes to network formation through endogenous transglutaminase activity and protein–protein interactions, while both insufficient structural modification and excessive aggregation may compromise gel integrity and functionality. These structural changes are relevant not only to gel functionality but also to digestion behaviour, because protein hydrolysis can be influenced by matrix organisation, network density, and enzyme accessibility as well as by composition alone [2,3].

Lipid fortification is commonly used to improve the nutritional profile of surimi products through incorporation of unsaturated oils. However, the introduction of lipid phases can influence both gel structure and oxidative stability. Oxidative behaviour in surimi is governed not only by lipid composition but also by the surrounding protein matrix, which affects oxygen diffusion, lipid accessibility, and interactions with endogenous pro-oxidants [4,5]. Previous studies have shown that lipid incorporation can alter gel properties, although the magnitude and direction of these effects depend on lipid type, physical state, and dispersion within the protein network [6,7,8].

High-pressure processing (HPP) is a non-thermal technology used to modify fish protein systems. Pressure-induced structural changes can promote protein unfolding and network formation, resulting in improvements in gel strength and water-holding capacity under appropriate processing conditions. However, excessive pressures may induce extensive aggregation and structural heterogeneity that reduce functional performance [9,10,11]. These responses are highly pressure-dependent, reflecting a balance between controlled unfolding and excessive aggregation. In addition to influencing gel structure, HPP may affect lipid accessibility, protein–lipid interactions, and oxidative behaviour through pressure-induced matrix restructuring.

Processing-induced structural changes are also increasingly recognised as important determinants of protein digestibility. Previous studies have shown that modifications to protein organisation, network density, and matrix composition can influence digestive behaviour by altering enzyme accessibility and hydrolysis pathways [3,12,13]. Alongside overall protein digestibility measurements, analysis of free amino acid release provides additional information on the products formed during gastrointestinal proteolysis [14].

Hoki (Macruronus novaezelandiae) is a commercially important New Zealand fish species that underpins one of the country’s largest finfish fisheries [15,16]. Hoki also has a documented history of commercial surimi production in New Zealand [16]. Its commercial relevance and previous utilisation for surimi manufacture make hoki an appropriate raw material for investigating the effects of processing and oil-fortification on structured fish protein systems.

Previous studies have examined the effects of lipid incorporation, HPP, and processing-related structural changes on surimi quality and digestion-related behaviour. However, it remains less clear how pressure level and oil fortification influence the same surimi system across technological properties, oxidative stability, microstructure, and digestion-related FAA release. The contribution of the present study is therefore the evaluation of pressure level and oil fortification within the same factorial framework, allowing their individual and combined effects across these linked responses to be assessed.

Accordingly, this study evaluated the combined effects of high-pressure processing and lipid fortification on the physicochemical, structural, oxidative, and digestion-related properties of hoki surimi. Surimi systems containing canola oil, flaxseed oil, or a no-added-oil control were subjected to a range of pressure treatments, and their technological properties, colour characteristics, lipid oxidation, microstructure, thermal behaviour, and free amino acid release during in vitro digestion were assessed to examine pressure–oil-fortification interactions in structured fish protein gels.

## 2. Materials and Methods

### 2.1. Materials and Experimental Design

Snap-frozen hoki fillets were purchased from a local supermarket (Sealord™, Nelson, New Zealand) and stored at −20 °C until use. Food-grade canola oil and flaxseed oil were purchased from Countdown Supermarket, Auckland, New Zealand.

The main experiment followed a factorial structure based on two factors: pressure level and oil fortification. Pressure levels were 0, 200, 300, 400, 500, and 600 MPa, and oil fortification treatments comprised a no-added-oil control, prepared with water in place of oil, canola oil, and flaxseed oil. Because the letter C was assigned to canola oil, the no-added-oil control was coded as R, derived from “Regular”, in sample labels and figures. Thus, “Regular” or “R” denotes the no-added-oil water control. Samples were coded according to oil-fortification treatment and pressure level as R-0 to R-600 for the no-added-oil control, C-0 to C-600 for canola oil, and F-0 to F-600 for flaxseed oil. For each oil-fortification × pressure treatment, three independently prepared formulation batches were produced. These three independently prepared batches constituted the sample-level replicates for quantitative analysis (*n* = 3). Where repeated analytical measurements were performed, these were treated as technical replicates and did not increase the independent sample size.

### 2.2. Surimi Preparation and Lipid Fortification

Frozen hoki fillets were partially thawed at room temperature for 10 min, cut into small cubes, and manually trimmed to remove visible connective tissue. The fish was processed into a coarse paste using a Braun MultiQuick 5 hand blender (stick-blender attachment; Braun Household, Neu-Isenburg, Germany) for 5 min. Sugar and NaCl were added at 2% and 3% (*w*/*w*), respectively, relative to the fish-paste mass, and the paste was blended using the same blender for a further 5 min to obtain a fine surimi paste. For selected unsalted samples prepared for DSC analysis, sugar was maintained at 2% (*w*/*w*), while NaCl was omitted.

For lipid fortification, canola oil or flaxseed oil was incorporated into the surimi paste at 10% (*v*/*w*) and homogenised for 5 min using the same blender to promote dispersion within the protein matrix. Control samples were prepared by adding water at the same level, 10% (*v*/*w*), in place of oil. The formulated pastes were portioned into 25 g blocks, vacuum-sealed in high-density polyethylene (HDPE) bags, and stored at −20 °C prior to further processing.

### 2.3. High-Pressure Processing and Thermal Gelation

Before thermal gelation, the formulated, vacuum-packaged surimi paste was subjected to high-pressure processing (HPP) at FoodBowl Ltd., Auckland, New Zealand. Samples were loaded into the pressure vessel and treated for 15 min at designated pressure levels of 200, 300, 400, 500, and 600 MPa. Unpressurised samples served as the 0 MPa control. The pressure series was selected to examine pressure-dependent changes across a broad treatment range, with 100 MPa increments from 200 to 600 MPa allowing pressure-dependent trends to be assessed. A fixed holding time of 15 min was used so that pressure level could be varied while treatment duration remained constant. A Multivac 55 L system (Multivac Sepp Haggenmüller GmbH & Co., Wolfertschwenden, Germany) was used for pressure treatment.

After pressure treatment, vacuum-packaged surimi samples were thermally gelled using a two-step water-bath process: 60 °C for 1 h, followed by 90 °C for 20 min. Cooked gels were stored at −20 °C for no longer than 1 week, until all HPP-processed samples were ready for subsequent testing, and were thawed overnight at 4 °C before analysis.

### 2.4. Proximate Composition

Moisture content was determined by oven drying according to AOAC 950.46B. Approximately 25 g of cooked surimi gel was weighed into a crucible and dried at 105 °C for 12 h in a convection oven (SANYO Electric Biomedical Co., Ltd., Osaka, Japan), cooled in a desiccator, and weighed. Moisture content was calculated gravimetrically.

Crude protein content was determined using the Kjeldahl method according to AOAC 928.08. Approximately 1 g of thawed sample was digested with potassium sulfate, copper sulfate, and concentrated sulfuric acid at 400 °C using a Velp DK 20 digester (VELP Scientifica Srl, Usmate, Italy). Distillation and titration were performed using a VAPODEST 450 system (Gerhardt GmbH & Co. KG, Königswinter, Germany). Protein content was calculated using a nitrogen-to-protein conversion factor of 6.25.

Total fat content was determined by Soxhlet extraction according to AOAC 991.36. Approximately 5 g of oven-dried sample was extracted with petroleum ether, 100 mL, using a Soxhlet apparatus for 8 h. Residual solvent was removed by drying at 105 °C for 1 h, followed by cooling in a desiccator and weighing. Fat content was calculated gravimetrically and expressed on a wet-weight basis using measured moisture content:

$$\mathrm{Fat}\;(\%)=\frac{m_{1}-m_{2}}{m_{1}}\times [100-\mathrm{moisture}\;\mathrm{content}\;(\%)]$$

where $m_{1}$ is the sample mass before Soxhlet extraction and $m_{2}$ is the sample mass after extraction.

Ash content was determined from oven-dried samples according to AOAC 920.153. Samples were combusted at 550 °C for 6 h in a muffle furnace (Model 200, McGregor Kiln Furnace, Auckland, New Zealand), cooled in a desiccator, and weighed. Carbohydrate content was calculated by difference from measured proximate components and was excluded from statistical modelling because it was a derived variable.

### 2.5. Technological Properties

Water-binding capacity (WBC) was determined using a centrifugation/filter-paper method adapted from established approaches for assessing water-holding properties of protein gels [17]. Approximately 2 g of cooked surimi gel was placed in a 15 mL centrifuge tube lined with two layers of filter paper and centrifuged at 3000× *g* for 10 min at 20 °C using a Centrifuge 5810R (Eppendorf AG, Hamburg, Germany). Samples were weighed before and after centrifugation, and WBC was calculated from sample mass loss corrected for initial moisture content:

$$\mathrm{WBC}\;(\%)=\left[1-\frac{m_{1}-m_{2}}{m_{1}\times (M/100)}\right]\times 100$$

where $m_{1}$ is the sample mass before centrifugation and $m_{2}$ is the sample mass after centrifugation, and *M* is the initial moisture content of the sample expressed as a percentage (%).

Cooking loss was determined from the difference in sample mass before and after thermal gelation, using an approach consistent with surimi gel studies in which expelled liquid is removed before post-cooking weighing [18]. Frozen surimi samples were thawed overnight at 4 °C before cooking. Thawed surimi portions were weighed before cooking ($m_{1}$), subjected to the thermal gelation procedure described above, removed from the packaging, gently blotted with filter paper to remove surface exudate, and reweighed ($m_{2}$). Cooking loss was calculated as:

$$\mathrm{Cooking}\;\mathrm{loss}\;(\%)=\frac{m_{1}-m_{2}}{m_{1}}\times 100$$

Measurements were conducted using three independently prepared sample packages for each treatment.

Texture was assessed as maximum slice shear force, defined as the maximum force recorded during shearing of the cooked gel sample, using a TA.XT plus texture analyser (Stable Micro Systems, Godalming, UK) at Massey University, Palmerston North, using a method adapted from Zhu et al. [19]. Cooked gel samples were cut into rectangular sections of approximately 4 × 4 × 1 cm and sheared using a flat blade attachment. The pre-test speed and test speed were both 1 mm/s, and the trigger force was 0.05 N. Measurements were recorded in triplicate for each treatment and expressed as slice shear force (N).

### 2.6. Colour Measurement

Colour was measured using a HunterLab/ColourFlex colourimeter (Hunter Associates Laboratory, Inc., Reston, VA, USA) calibrated with standard white, green, and black plates. Cooked gel samples were cut into small cubes and placed in a Petri dish for measurement. Colour was expressed as L* for lightness, a* for redness/greenness, and b* for yellowness/blueness. Measurements were performed in triplicate. Whiteness was calculated from L*, a*, and b* values using the following equation [12]:

$$\mathrm{Whiteness}=100-{\left[(100-L^{*}{)}^{2}+a^{*2}+b^{*2}\right]}^{1/2}$$

Whiteness was used as the summary colour-quality index because it reflects the lightness/whiteness characteristics relevant to surimi gels and integrates the measured L*, a*, and b* colour coordinates.

### 2.7. Lipid Oxidation by Thiobarbituric Acid Reactive Substances

Lipid oxidation was assessed using the thiobarbituric acid reactive substances (TBARS) assay, based on the reaction of thiobarbituric acid with malondialdehyde (MDA) and related aldehydic oxidation products to form a pink chromogen measurable at approximately 531–535 nm [20,21,22]. Approximately 5 g of surimi sample was homogenised with 15 mL deionised water using an Ultra-Turrax T25 Basic homogeniser (IKA-Werke GmbH & Co. KG, Staufen, Germany) at 14,000 rpm for 30 s. A 1 mL aliquot of homogenate was mixed with 50 μL butylated hydroxytoluene solution, 7.2% (*w*/*v*) in ethanol, and 2 mL TBA–TCA reagent, consisting of 20 mM thiobarbituric acid in 15% (*w*/*v*) trichloroacetic acid.

The mixture was vortexed, heated in a water bath at 95 °C for 15 min to allow colour development, cooled in an ice bath for 10 min, and centrifuged at 2000× *g* for 10 min at 4 °C using a Centrifuge 5810R (Eppendorf). The supernatant was further clarified by centrifugation at 10,000× *g* for 5 min using a Z 216 MK centrifuge (Hermle Labortechnik GmbH, Wehingen, Germany). Absorbance was measured at 531 nm using a UV–Vis spectrophotometer (Ultrospec 7000, GE Healthcare, Cambridge, UK). TBARS values were calculated using the Beer–Lambert relationship and expressed as mg MDA equivalents per kg sample.

TBARS analysis was conducted in triplicate at 0, 300, and 500 MPa only. Owing to analytical time and operational constraints, these treatments were selected as a subset representing unpressurised, intermediate-, and higher-pressure conditions. Statistical analysis and interpretation were therefore restricted to this measured subset and were not used to infer a complete pressure–response relationship. Because the TBARS assay is not specific to MDA and can respond to other TBA-reactive compounds, values were interpreted as relative indicators of oxidative status rather than absolute MDA concentrations [21].

### 2.8. Scanning Electron Microscopy

Cross-sectional samples of approximately 2 × 2 × 2 mm were cut from selected surimi gels and freeze-dried for 48 h using an ALPHA 2–4 LDplus freeze dryer (Martin Christ, Osterode am Harz, Germany). Dried samples were mounted on aluminium stubs and sputter-coated with platinum for 40 s using a Hitachi E-1045 sputter coater (Hitachi High-Technologies Corporation, Tokyo, Japan). Microstructural observations were performed using a Hitachi SU-70 Schottky field emission scanning electron microscope (Hitachi High-Technologies Corporation, Tokyo, Japan) operated at an accelerating voltage of 5 kV. Images were acquired at low magnification, approximately ×300–×450, and at high magnification, ×3000, to examine overall gel network structure and fine microstructural features. Micrographs were used for qualitative comparison of selected oil fortification treatments and pressure treatments.

### 2.9. In Vitro Digestion

In vitro digestion was conducted using a static oral–gastric–intestinal model with electrolyte compositions reported previously for simulated gastrointestinal digestion [23] and with conceptual alignment to the INFOGEST static digestion framework [24]. Simulated salivary fluid (SSF), simulated gastric fluid (SGF), and simulated intestinal fluid (SIF) were prepared from electrolyte stock solutions. A 0.3 mol/L CaCl_2_·2H_2_O stock solution was prepared separately for phase-specific calcium addition, while 1 and 6 mol/L HCl and 1 mol/L NaOH solutions were prepared for pH adjustment. Enzyme and bile solutions were prepared as 1.0% (*w*/*v*) α-amylase in SSF, 10.0% (*w*/*v*) pepsin in SGF, 1.07% (*w*/*v*) pancreatin in SIF, and 3.84% (*w*/*v*) bile in deionised water. All digestion solutions were stored at 4 °C before use. Digestion reagents included α-amylase from Aspergillus oryzae, pepsin from porcine gastric mucosa, pancreatin, porcine bile extract, and the commercial lipase preparation Lipozyme TL 100 L (Novozymes).

For the oral phase, 5 g of minced surimi was combined with 3.5 mL SSF, 25 µL CaCl_2_ stock solution, and 975 µL deionised water in a Schott bottle. The components were mixed, the pH was adjusted to 7, and the sample was equilibrated in a shaking water bath (Gyrotory G76, New Brunswick Scientific Co., Inc., Edison, NJ, USA) at 37 °C and 5 rpm. Oral digestion was initiated by adding 0.5 mL α-amylase solution, providing a final α-amylase activity of approximately 75 U/mL, and continued for 5 min.

For the gastric phase, 7.5 mL SGF, 5 µL CaCl_2_ stock solution, 695 µL deionised water, and 0.2 mL 1 mol/L HCl were added to the oral digest. The pH was adjusted to 3 using 6 mol/L HCl, after which 1.6 mL pepsin solution was added, providing a final pepsin activity of approximately 2000 U/mL, and digestion continued for 2 h at 37 °C and 5 rpm. At the end of gastric digestion, a 2 mL aliquot was collected and snap-frozen in liquid nitrogen to stop further enzymatic activity.

For the intestinal phase, 11 mL SIF, 40 µL CaCl_2_ stock solution, 1.31 mL deionised water, and 0.15 mL 1 mol/L NaOH were added to the remaining gastric digest. The pH was adjusted to 7 using 1 mol/L NaOH, after which 5 mL pancreatin solution, 2.5 mL bile solution, and 780 µL Lipozyme TL 100 L were added. The pancreatin addition provided a final trypsin activity of 100 U/mL. Intestinal digestion was continued for 3 h at 37 °C and 5 rpm. At the end of digestion, a 5 mL aliquot was collected and snap-frozen in liquid nitrogen.

After thawing, the gastric and intestinal aliquots were centrifuged at 2000× *g* for 10 min at 4 °C using a Centrifuge 5810R (Eppendorf). A 1.2 mL portion of each supernatant was transferred to a microcentrifuge tube and further centrifuged at 10,000× *g* for 5 min at 4 °C using a Z 216 MK centrifuge (Hermle Labortechnik GmbH, Germany). A 1 mL portion of each clarified supernatant was transferred to a clean tube and stored at −20 °C until FAA analysis. Three independent formulation batches were prepared for each treatment as described in Section 2.1. The digestion experiment was conducted in three separate runs (*n* = 3).

### 2.10. Free Amino Acid Extraction and LC–MS/MS Analysis

Free amino acids (FAAs) present before digestion were extracted using a modified methanolic procedure previously reported for FAA analysis [25]. Briefly, 1 g of surimi sample was combined with 7.5 mL of HPLC-grade methanol and 5.4 g of glass beads, 2 mm in diameter, in a 50 mL centrifuge tube. The mixture was vortexed at high speed for 30 min using a Vortex-Genie 2 mixer (Scientific Industries, Inc., Bohemia, NY, USA). The pH was adjusted to 3.5–4.5, and the sample was allowed to stand until layer separation occurred. A 1.2 mL aliquot of the upper layer was transferred to a microcentrifuge tube and centrifuged at 10,000× *g* for 10 min at 4 °C using a Z 216 MK centrifuge (Hermle Labortechnik GmbH, Germany). The clarified supernatant was transferred to a clean microcentrifuge tube and stored at −20 °C until analysis. Three independent formulation batches were prepared per treatment, and FAA extraction before digestion was performed on three sample-level replicates per treatment (*n* = 3). Gastric- and intestinal-phase samples were collected and clarified as described in the In Vitro Digestion section.

FAA derivatisation was performed using a modified AccQ-Tag procedure [26], while the subsequent LC–MS/MS workflow was informed by previously reported amino acid profiling conditions [27]. Reagents included 200 mM sodium tetraborate buffer containing 5% acetonitrile at pH 8.8, internal-standard-spiked methanol containing 10 mg/L d_4_-alanine, neutralising solution, and AccQ-Tag reagent prepared by dissolving 2.8 mg of 6-aminoquinolyl-N-hydroxysuccinimidyl carbamate in 1 mL of dry acetonitrile. A five-point calibration series was prepared by serial dilution of an amino acid standard. Samples collected before digestion and after the gastric and intestinal phases were diluted tenfold with LC–MS-grade methanol. A 40 µL aliquot of diluted sample was mixed with 40 µL of internal-standard-spiked methanol, vortexed, and centrifuged at 10,000× *g* for 5 min at 4 °C. For derivatisation, 70 µL of borate buffer and 10 µL of sample, standard, or blank were transferred to a 0.7 mL polypropylene shell vial, followed by 10 µL of AccQ-Tag reagent. The vials were immediately capped, vortexed, and incubated at 55 °C for 15 min. After incubation, 400 µL of neutralising solution was added, and the derivatised samples were stored at −20 °C until analysis.

LC–MS/MS analysis was performed using an Agilent 1260 Infinity HPLC system coupled to an Agilent 6420 triple-quadrupole LC/MS system (Agilent Technologies, Inc., Santa Clara, CA, USA). Chromatographic separation was achieved using a Kinetex C18 core–shell column, 100 × 2.1 mm, 1.7 µm. Detection was performed using positive-mode electrospray ionisation with multiple-reaction monitoring. Amino acid concentrations were determined from calibration curves using d_4_-alanine as the internal standard. Data were processed using MassHunter Qualitative Analysis (B.06.00) and MassHunter QQQ Quantitative Acquisition software (B.07.00) and expressed as mg/kg sample. For digestion-phase samples, the total digest volume at each sampling point was recalculated, and FAA concentrations were adjusted accordingly before being expressed as mg/kg sample.

Total FAA concentration was calculated as the sum of the quantified individual amino acids within each digestion phase. The digestion analysis focused on protein hydrolysis in relation to HPP treatment; therefore, FAA release was used as a protein-focused digestion endpoint. FAA concentrations were interpreted as comparative indicators of relative protein hydrolysis across treatments rather than absolute measures of total protein digestibility, because peptides and oligopeptides were not quantified and also contribute to digestible protein and amino acid availability [28,29].

### 2.11. Differential Scanning Calorimetry

Differential scanning calorimetry (DSC) was performed on selected representative surimi samples to examine thermal transitions associated with pressure treatment and salt addition. Representative samples included no-salt unpressurised surimi, no-salt surimi treated at 300 MPa, and surimi prepared with 3% (*w*/*w*) NaCl relative to the fish-paste mass. Approximately 10–15 mg of thawed sample was sealed in a hermetic aluminium pan and analysed using a TA Instruments DSC Q2000 equipped with an RCS40 cooling system (TA Instruments, New Castle, DE, USA) at the Riddet Institute, Massey University, Palmerston North, New Zealand, using a modified procedure reported previously [10]. An empty sealed pan was used as reference. Samples were heated from 20 °C to 105 °C at 5 °C/min under nitrogen. Thermograms were used for descriptive comparison rather than quantitative thermal analysis because the DSC assessment was limited to selected representative samples. The DSC analysis was not designed as a factorial assessment of oil-fortification effects.

### 2.12. Sodium Dodecyl Sulfate–Polyacrylamide Gel Electrophoresis

Protein profiles of selected surimi samples were evaluated by one-dimensional sodium dodecyl sulfate–polyacrylamide gel electrophoresis (SDS-PAGE) under reducing conditions using NuPAGE™ 10% Bis-Tris Midi Protein Gels (Life Technologies New Zealand Limited, Auckland, New Zealand). Selected samples represented untreated surimi (0 MPa), pressure-treated surimi (300 and 600 MPa), and salted surimi prepared with 3% (*w*/*w*) NaCl relative to the fish-paste mass. Samples were mixed with reducing SDS sample loading buffer containing Tris-HCl, SDS, 2-mercaptoethanol, glycerol, and bromophenol blue and diluted to obtain a protein concentration of approximately 4 mg/mL. Prepared samples were heated at 100 °C for 5 min and centrifuged at 10,000 rpm for 5 min. Approximately 40 µg protein was loaded per well, and a Novex™ Sharp Pre-stained Protein Standard (3.5–260 kDa; Life Technologies New Zealand Limited) was used as the molecular-weight marker. Electrophoresis was performed in 1× NuPAGE^®^ MES SDS running buffer at a constant voltage of 150 V for 1 h. Gels were stained with Coomassie Brilliant Blue G-250 and scanned using a GS-900™ calibrated densitometer (Bio-Rad Laboratories, Inc., Hercules, CA, USA). Gel profiles were analysed using Image Lab™ software (version 5.2.1, Bio-Rad) [30]. The selected-sample SDS-PAGE analysis was intended as a qualitative comparison of pressure- and salt-associated protein banding patterns and was not designed as a factorial assessment of oil-fortification effects.

### 2.13. Statistical Analysis

Statistical analyses were performed using R, version 4.3.3, and RStudio, version 2026.01.2.418. A significance level of *p* < 0.05 was applied throughout. Proximate composition, cooking loss, WBC, texture, colour variables (L*, a*, b*, and whiteness), and TBARS were analysed using two-way analysis of variance (ANOVA), implemented through fixed-effect linear models with oil fortification, pressure level, and their interaction as model terms. Carbohydrate content was excluded from statistical modelling because it was calculated by difference. TBARS analysis was conducted in triplicate on selected pressure treatments, 0, 300, and 500 MPa, as representative pressure levels for assessing lipid oxidation. Therefore, statistical analysis for TBARS was restricted to this measured pressure subset.

FAA data were analysed using fixed-effect linear models to evaluate the effects of digestion phase, oil fortification, and pressure level. For intestinal-phase total FAA, oil fortification, pressure level, and their interaction were evaluated. Because the same samples were followed sequentially through the digestion stages, mixed-effects models incorporating sample identity as a random effect were initially explored to account for repeated measurements across phases. However, the random-effect contribution was negligible and did not improve model fit; therefore, fixed-effect linear models were retained. For comparisons involving digestion phase, analyses were restricted to treatment combinations represented across the relevant phases. The 200 MPa canola- and flaxseed-oil treatments were available before digestion but were not represented in the gastric or intestinal datasets and were therefore excluded from phase-comparison models. Total FAA was calculated as the sum of quantified individual FAAs within each digestion phase. Where significant effects were identified, pairwise comparisons were performed using estimated marginal means. Heatmaps were generated from intestinal-phase FAA concentrations after scaling across amino acids. Results are presented as mean ± SD, *n* = 3, unless otherwise stated.

## 3. Results and Discussion

### 3.1. Proximate Composition

Moisture, fat, ash, and protein contents of surimi samples prepared with different oil-fortification treatments and pressure treatments are presented in Table 1. Moisture content was significantly influenced by oil fortification, with the no-added-oil control showing higher values (71.9–73.3%) than canola oil (65.2–66.4%) and flaxseed oil treatments (64.5–65.9%). No clear pressure-dependent trend was observed within each oil-fortification treatment. The higher moisture content of the control reflected the absence of added lipid, whereas oil incorporation reduced the relative water fraction within the matrix, consistent with previous reports in surimi systems [31,32]. This indicates that the main compositional difference among samples was introduced by the oil-fortification step rather than by pressure processing.

Fat content also differed mainly according to oil fortification. Canola and flaxseed oil treatments showed significantly higher fat contents (8.52–9.29%) than the no-added-oil control (1.24–1.70%), directly reflecting the 10% oil fortification step. Pressure treatment did not significantly affect measured fat content, indicating that HPP did not change the overall lipid level measured by proximate analysis. This is important for interpreting later oxidative and structural responses, because pressure-related differences in TBARS, water binding, or microstructure cannot be explained by changes in total lipid quantity alone, but more likely relate to changes in lipid distribution, lipid accessibility, and protein–lipid organisation within the gel matrix.

Ash content showed moderate variation across treatments (3.20–4.13%), while protein content remained broadly similar (12.99–15.03%). The two-way ANOVA indicated significant pressure and interaction effects for ash and significant oil fortification and interaction effects for protein. However, the small magnitude and inconsistent direction of these changes suggest that they should be interpreted cautiously rather than as evidence of a consistent pressure-driven change in mineral or protein content. These variations are more likely associated with compositional redistribution effects, such as moisture–lipid balance and concentration changes, rather than true changes in mineral or protein content.

The corresponding two-way ANOVA results for proximate composition are summarised in Table 2. Carbohydrate was excluded from statistical modelling because it was calculated by difference.

Overall, proximate composition was influenced mainly by oil fortification, especially through the increase in fat content and corresponding reduction in relative moisture content in oil-fortified samples. Pressure effects were limited at the bulk compositional level, although ash and protein showed significant interaction effects that were small in magnitude and inconsistent in direction. These findings suggest that lipid fortification predictably changed the moisture–fat balance of the surimi system, whereas HPP did not substantially alter bulk composition. This compositional baseline is important because subsequent changes in water-binding capacity, cooking loss, texture, colour, oxidative stability, microstructure, and FAA release are unlikely to be explained by proximate composition alone. Instead, they should be interpreted mainly in relation to pressure-induced matrix restructuring and protein–lipid interactions.

### 3.2. Technological Properties

Cooking loss, water-binding capacity (WBC), and texture, measured as slice shear force, of surimi samples prepared with different oil-fortification treatments and pressure treatments are presented in Table 3. Cooking loss was lower in pressure-treated samples than in the 0 MPa samples, with the highest values observed at 0 MPa (6.58–7.39%) and lower values at 400–600 MPa (3.46–4.93%). At comparable pressure levels, control samples showed numerically higher cooking loss than oil-fortified samples, although the main reduction was associated with pressure treatment. This pattern, together with the WBC response, suggests that pressure treatment improved water retention and reduced thermal exudation, consistent with pressure-induced modification of myofibrillar protein structure reported in surimi systems [10,11]. This is practically relevant because lower cooking loss indicates improved product yield and reduced fluid release during thermal gelation.

The corresponding two-way ANOVA results for technological properties are summarised in Table 4. Cooking loss, water-binding capacity, and texture were analysed using oil fortification, pressure, and their interaction as model terms.

Cooking loss was affected by oil fortification and pressure, but not by their interaction (Table 4), indicating that the reduction in cooking loss can be interpreted mainly through separate oil-fortification and pressure effects.

WBC was affected by oil fortification, pressure, and their interaction (Table 4). WBC generally increased with pressure up to 500 MPa, followed by a slight decline at 600 MPa. Canola samples showed an increase from 76.99% at 0 MPa to 83.47% at 500 MPa, while flaxseed and control samples followed similar trends at lower absolute values. Oil-fortified samples generally exhibited higher WBC than the control, suggesting that lipid fortification influenced water retention behaviour. The non-linear pressure response indicates that oil incorporation altered network organisation and water distribution within the gel system [31,32,33]. This suggests that the dispersed lipid phase contributed to the gel matrix rather than acting only as added fat, thereby modifying water immobilisation. The higher absolute WBC values in canola samples indicate that the effect of lipid fortification depended on how the oil phase interacted with the protein network, rather than on oil addition alone.

Texture, measured as maximum slice shear force, was affected mainly by pressure, with a weaker oil-fortification effect and no oil fortification × pressure interaction (Table 4). Slice shear force increased from 64.26–65.64 N at 0 MPa to 68.41–70.53 N at 400–600 MPa. This trend was consistent across oil-fortification treatments, with only minor differences in magnitude. These results indicate that pressure-induced structural modification of the protein network was the primary driver of texture development, consistent with reported effects of HPP on surimi gel formation [11,33]. The increase in slice shear force suggests that pressure treatment strengthened the gel matrix, most likely through enhanced protein unfolding, intermolecular association, and network consolidation during subsequent heating. However, the slight stabilisation or decline in WBC at higher pressures suggests that greater pressure-associated compaction did not necessarily result in proportional functional improvement across all parameters.

Overall, the functional results indicate that pressure treatment improved cooking stability, water retention, and texture development, while oil fortification had a more specific effect on water-binding behaviour. The response was not simply linear, because WBC declined slightly at 600 MPa despite the general improvement at moderate pressure levels. These findings suggest that the most useful pressure range is likely one that promotes matrix consolidation without excessive compaction.

### 3.3. Colour Properties

Lightness (L*), redness/greenness (a*), yellowness/blueness (b*), and whiteness of surimi samples prepared with different oil-fortification treatments and pressure treatments are presented in Table 5. Colour properties were influenced mainly by oil fortification. Pressure effects were smaller than oil-fortification effects for L* and whiteness and were not significant for a*, whereas b* showed a significant oil fortification × pressure interaction. Flaxseed oil samples showed the highest L* values, 78.77–79.50, followed by canola oil, 76.74–77.40, while the no-added-oil control showed lower values, 71.63–72.21. Whiteness followed the same general pattern, with flaxseed oil > canola oil > control. This indicates that lipid fortification had a stronger effect on the visual appearance of the gels than pressure treatment.

The corresponding two-way ANOVA results for colour properties are summarised in Table 6.

The a* values were more negative in control samples, −3.52 to −3.11, than in canola and flaxseed oil treatments, approximately −2.60 to −1.90, indicating a lower red colour coordinate in the absence of added lipid. Yellowness was strongly oil-fortification-dependent, with flaxseed oil samples showing the highest b* values, 9.59–10.39, followed by canola oil, 8.00–8.78, while the control remained lower, 3.01–3.98. The significant oil fortification × pressure interaction for b* (Table 6) indicates that pressure effects on yellowness were oil-dependent, with greater fluctuation in control samples and relatively stable high values in oil-fortified samples. However, the magnitude and consistency of the L* and whiteness differences suggest that the incorporated oil phase was more important than pressure level in determining overall colour appearance.

The higher L*, whiteness, and b* values in oil-fortified samples suggest that lipid incorporation altered the optical properties of the gel matrix, likely through increased light scattering from the dispersed lipid phase and the intrinsic colour contribution of the oils, particularly flaxseed oil, consistent with previous reports [31,32]. In contrast, the lower L* values of control samples may reflect reduced light scattering and greater visibility of underlying pigments in the protein matrix. This is relevant to product quality because colour and whiteness are practical quality attributes in surimi-based products. The limited effect of HPP on most colour parameters suggests that pressure treatment improved technological properties without causing major undesirable colour shifts. These results support the interpretation that oil fortification modified the matrix environment and optical behaviour, while pressure acted mainly through structural and water-binding mechanisms rather than through strong changes in gel colour.

### 3.4. Lipid Oxidation (TBARS)

Lipid oxidation was assessed using thiobarbituric acid reactive substances (TBARS), and values for the selected pressure treatments, 0, 300, and 500 MPa, are presented in Table 7. Two-way ANOVA showed that TBARS values were significantly affected by oil fortification, pressure, and their interaction (*p* < 0.001), with the analysis restricted to the measured pressure subset. Clear oil-dependent differences were observed, with flaxseed oil samples showing the highest values, 1.114–1.262 mg MDA/kg, followed by canola oil, 1.009–1.151 mg MDA/kg, while the no-added-oil control showed substantially lower values, 0.506–0.546 mg MDA/kg. Across comparable pressure levels, oil-fortified samples showed higher TBARS values than the control, indicating that lipid fortification increased oxidative susceptibility. Together with the functional and colour results, these findings indicate that oil fortification not only modified the physical properties of the surimi matrix but also introduced a more oxidation-sensitive lipid phase.

Within each oil-fortification treatment, TBARS values decreased from 0 to 500 MPa, although the magnitude of reduction differed among oil-fortification treatments, resulting in a significant oil fortification × pressure interaction. This indicates that oxidation behaviour differed among oil-fortification treatments rather than changing uniformly with pressure. The higher TBARS values in flaxseed oil samples are consistent with the greater susceptibility of polyunsaturated lipids to peroxidation [7], whereas canola oil samples showed comparatively lower oxidation. The lower values in the control further indicate that the absence of added lipid limited oxidation within the system. Therefore, the oxidative stability of fortified surimi gels depended strongly on the lipid phase introduced into the matrix.

The decrease in TBARS with pressure suggests that pressure-associated matrix restructuring may have altered lipid accessibility and oxidation behaviour in an oil-dependent manner [10]. This pressure effect should not be interpreted simply as HPP preventing oxidation, because TBARS was measured only at selected pressure levels and the assay reflects TBA-reactive compounds rather than all oxidation products. A more cautious interpretation is that pressure-induced changes in protein organisation, water immobilisation, and protein–lipid interactions may have influenced the formation, exposure, or extractability of TBA-reactive compounds under the conditions tested. This interpretation is consistent with the functional results, where pressure improved water retention and matrix consolidation, suggesting that oxidation behaviour was linked to the physical organisation of the gel rather than to lipid content alone.

Overall, TBARS values differed most clearly among oil-fortification treatments, while pressure appeared to affect oxidation indirectly through restructuring of the protein–lipid system. This highlights an important trade-off: lipid fortification can modify product functionality, but the choice of oil strongly affects oxidative stability. Flaxseed oil produced the highest TBARS values, indicating greater oxidation risk, whereas canola oil showed comparatively lower oxidation while still contributing to the oil-fortified matrix. These results further indicate that oxidative behaviour in fortified surimi is governed by both lipid composition and matrix organisation, rather than by added lipid level alone.

### 3.5. Free Amino Acid Profile

Free amino acid (FAA) release was influenced mainly by digestion phase, with phase identified as the dominant factor in the linear model analysis (*p* < 0.001). Oil fortification showed amino acid-dependent effects, whereas pressure effects were comparatively limited and non-linear. Representative amino acids, including lysine, leucine, alanine, glutamic acid, and proline, were selected to illustrate phase-dependent release patterns across essential, non-essential, and structure-related amino acids. FAA analysis was included because it provides a more specific view of digestion products than bulk digestibility alone, allowing comparison of how individual amino acids were released in free form during simulated digestion [28,29]. Because FAA analysis included digestion phase and amino acid-specific treatment patterns, the results are reported separately in text and figures rather than as part of the two-way ANOVA summary tables.

**Figure 1:**
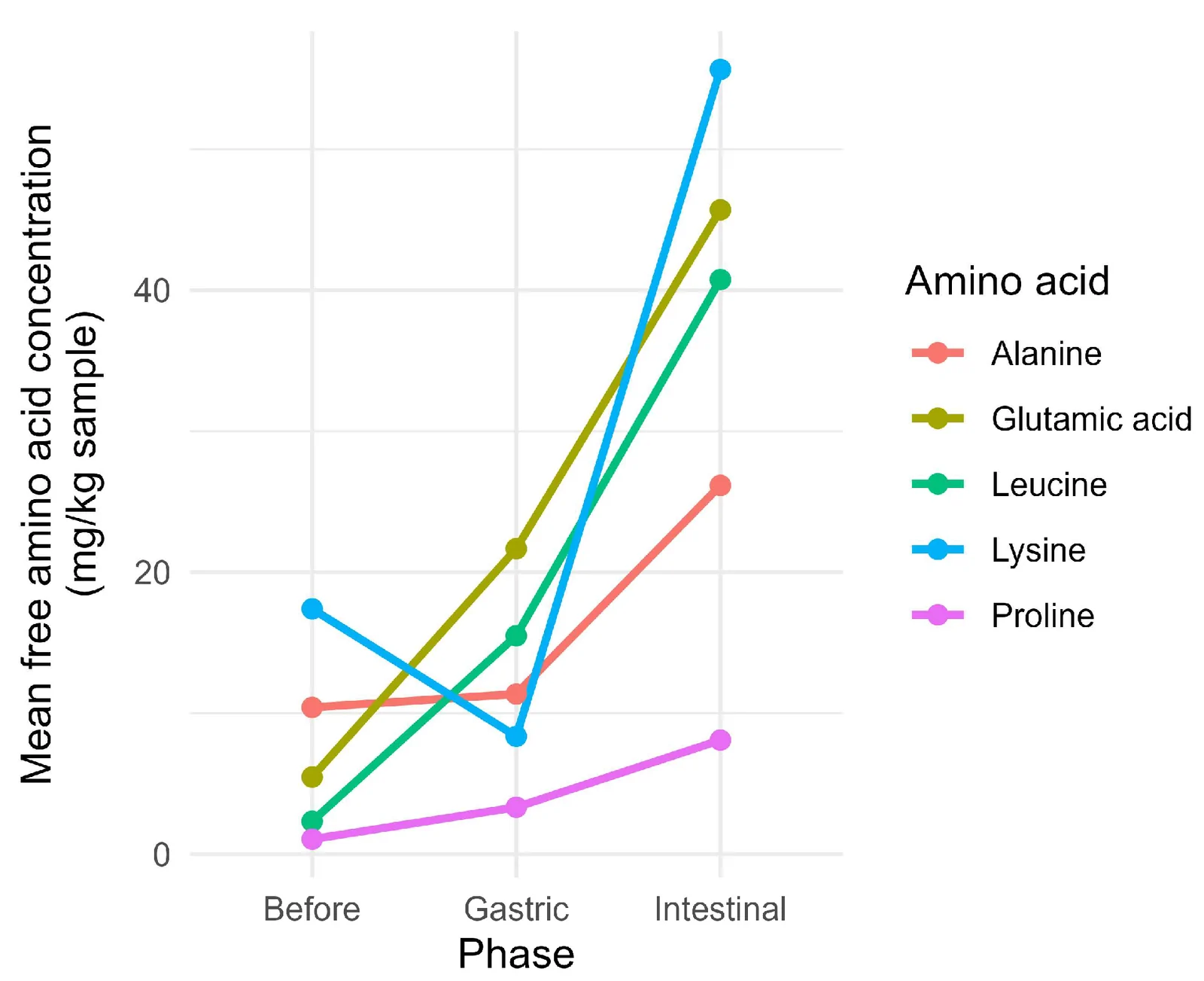
Phase-dependent changes in selected free amino acid concentrations across digestion phases. Values represent mean concentrations across treatments and are expressed as mg/kg sample.

Phase-dependent changes in selected FAA concentrations are shown in Figure 1. Most amino acids increased toward the intestinal phase, although release patterns differed among individual amino acids. Alanine increased progressively across digestion phases, while glutamic acid and leucine showed pronounced increases from the gastric to intestinal phase. Proline showed a modest increase. Lysine decreased during the gastric phase before increasing markedly in the intestinal phase, suggesting amino acid-specific differences in accessibility and release behaviour. Mechanistically, the increase in FAA concentration reflects progressive enzymatic hydrolysis of peptide bonds during digestion, with the larger intestinal-phase response indicating that pancreatic hydrolysis was more effective than gastric digestion in converting protein- and peptide-derived material into free amino acids.

**Figure 2:**
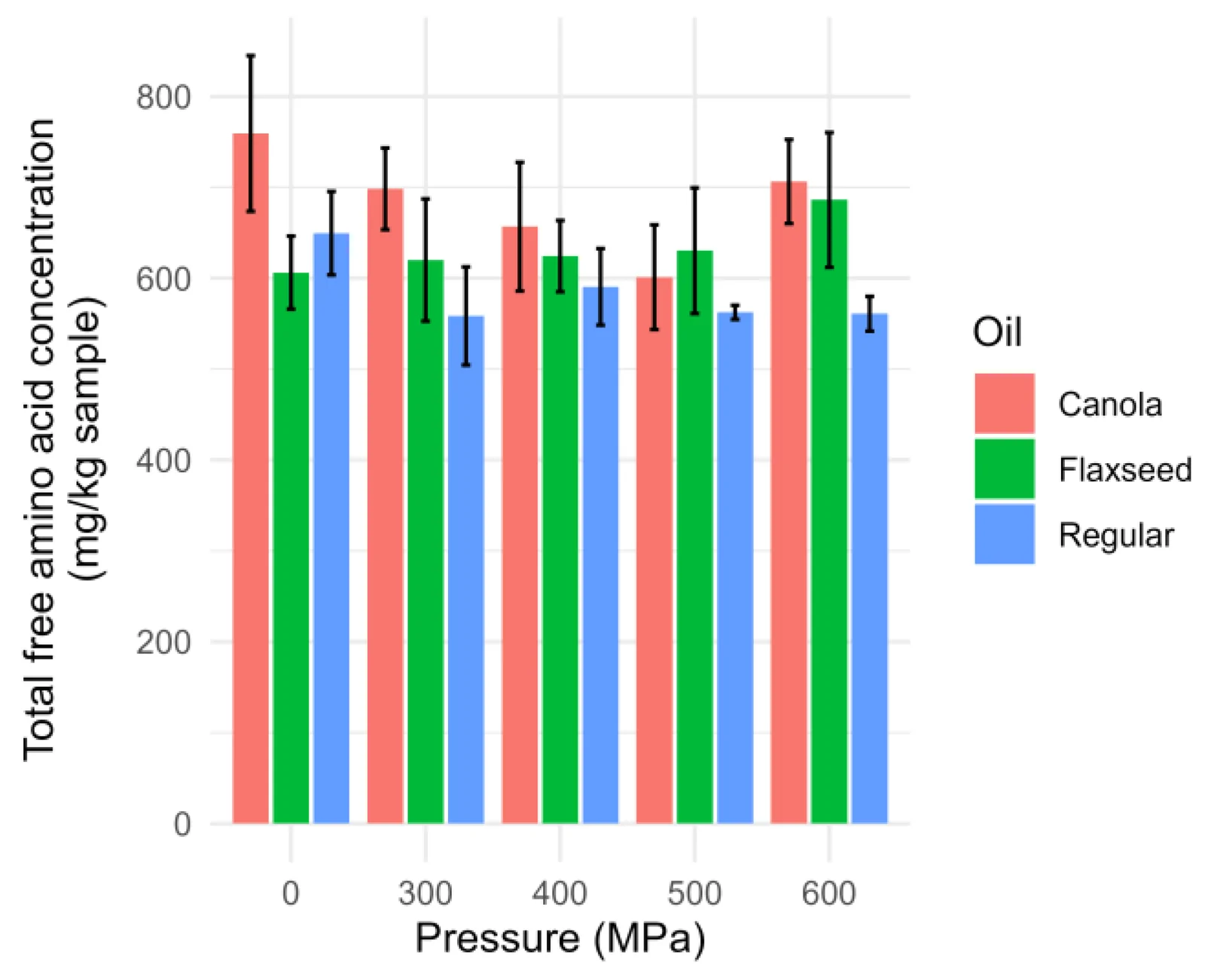
Total free amino acid concentration in the intestinal phase of hoki surimi samples as affected by oil fortification and pressure treatment (mean ± SD, *n* = 3). Values are expressed as mg/kg sample. Regular denotes the no-added-oil water control.

Total FAA concentration in the intestinal phase is shown in Figure 2. Canola oil samples generally showed higher total FAA concentrations, 601–759 mg/kg sample, followed by flaxseed oil, 606–686 mg/kg sample, while the no-added-oil control showed lower values, 558–650 mg/kg sample. Pressure effects were non-linear, with reduced FAA concentrations at some intermediate pressures and partial recovery at 600 MPa. These results indicate that oil fortification influenced FAA release, but pressure did not produce a uniform directional response across oil-fortification treatments. Therefore, HPP should not be interpreted as simply increasing FAA release; rather, pressure appeared to modulate enzyme accessibility in a matrix-dependent manner.

To compare total FAA concentrations across digestion stages, the phase-specific means were expressed relative to the sum of the three phase means (Table 8). The before-digestion, gastric-phase, and intestinal-phase means accounted for 7.2%, 18.1%, and 74.7%, respectively, with the intestinal phase showing the highest total FAA concentration. This pattern is consistent with continued protein and peptide hydrolysis during intestinal digestion [23,24]. The dominance of the intestinal phase also shows that the structured surimi matrix was not fully converted into free amino acids during the gastric stage, and that substantial hydrolysis occurred only after exposure to intestinal enzymes.

**Figure 3:**
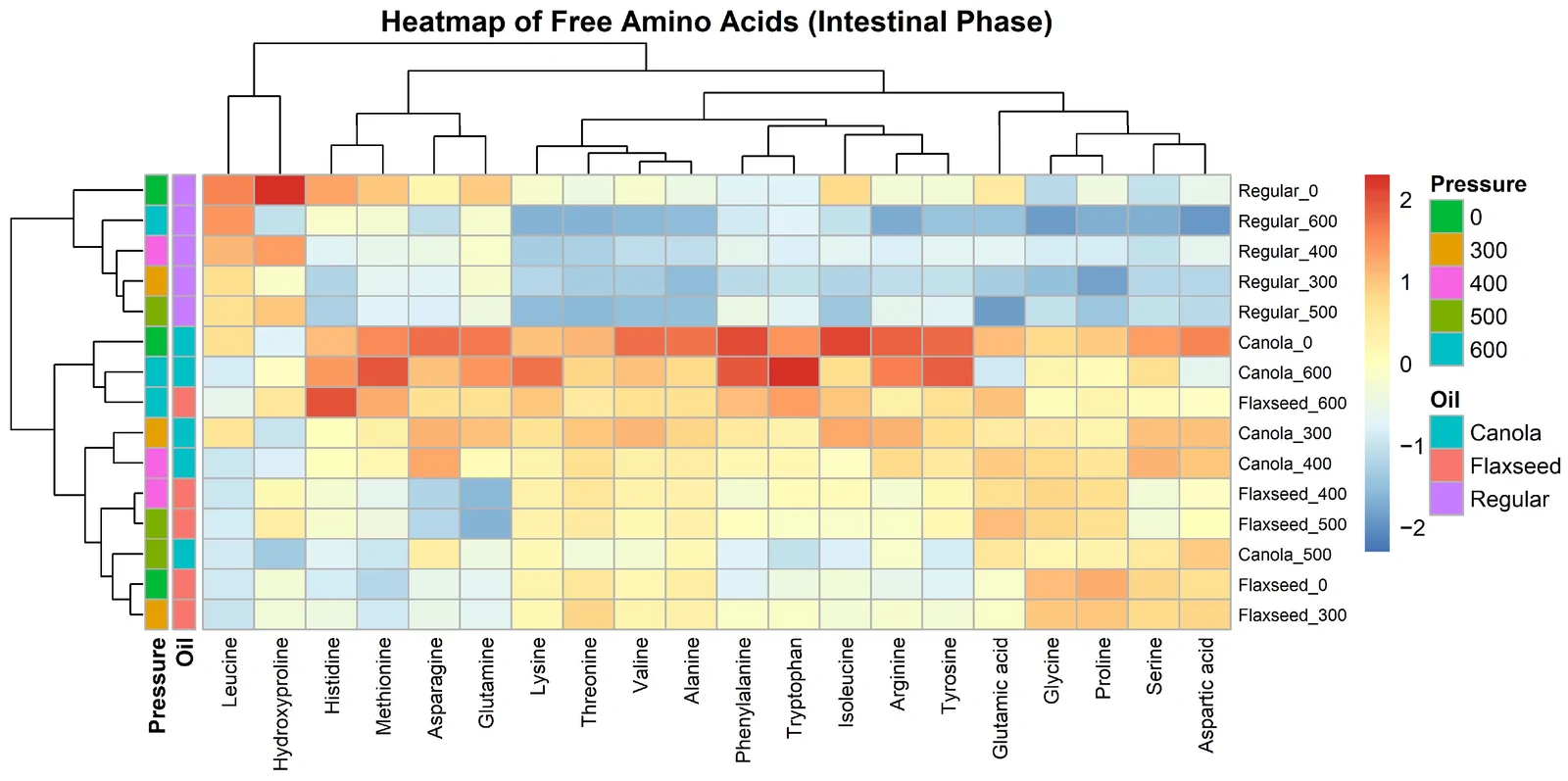
Heatmap showing scaled free amino acid profiles in the intestinal phase across oil fortification and pressure treatments. Values are scaled for visual comparison of relative FAA distribution patterns. Regular denotes the no-added-oil water control.

A heatmap of intestinal-phase FAA profiles is shown in Figure 3. The scaled profiles showed oil-fortification-dependent separation between the no-added-oil control and oil-fortified samples. In contrast, pressure levels did not form distinct clusters, indicating that pressure-related differences in intestinal-phase FAA profiles were comparatively limited and non-uniform. Because the heatmap presents scaled values, it was interpreted primarily as a comparison of relative profile patterns among treatments rather than absolute concentrations among individual amino acids. The heatmap therefore supports the interpretation that oil fortification had a clearer effect on the relative FAA profile than pressure level.

The phase-dependent increase in FAA concentration reflects progressive proteolysis during digestion. The marked intestinal-phase increases in glutamic acid, leucine, and alanine suggest release from peptide fractions that were less accessible during gastric digestion but more susceptible to pancreatic hydrolysis. The delayed increase in lysine indicates amino acid-specific accessibility within the protein matrix, while the modest proline response may reflect greater resistance to enzymatic cleavage. This variation is important because different amino acids were not released uniformly, indicating that digestion behaviour depended not only on total protein content but also on protein structure, peptide sequence, and matrix accessibility.

Oil fortification influenced FAA release, possibly through lipid–protein interactions that altered enzyme accessibility within the gel matrix; however, the absence of a consistent trend across oil-fortification treatments suggests that this effect depended on lipid composition and distribution [6,32]. Pressure effects were secondary and non-linear. Reduced FAA at some intermediate pressures may reflect pressure-associated aggregation or network compaction limiting enzyme accessibility, while partial recovery at higher pressure suggests structural rearrangement that partly restored accessibility, consistent with pressure-induced protein unfolding and aggregation behaviour [9,34]. These findings suggest that pressure modified digestion behaviour indirectly through structural changes in the gel matrix rather than through a simple increase in protein breakdown.

Overall, digestion phase was the primary determinant of FAA release, while oil fortification and pressure acted as secondary modifiers through matrix structure and accessibility effects. From a nutritional interpretation perspective, the release of essential amino acids such as leucine and lysine is relevant because it indicates that digestion generated free amino acid forms in vitro [28,29]. However, the present in vitro data should not be overinterpreted as evidence of specific health benefits or in vivo bioavailability, because peptide fractions, absorption, and physiological outcomes were not measured. Thus, FAA profiling showed treatment-dependent differences in digestion products and enzyme accessibility within a structured protein–lipid gel system.

### 3.6. Thermal Properties (DSC)

**Figure 4:**
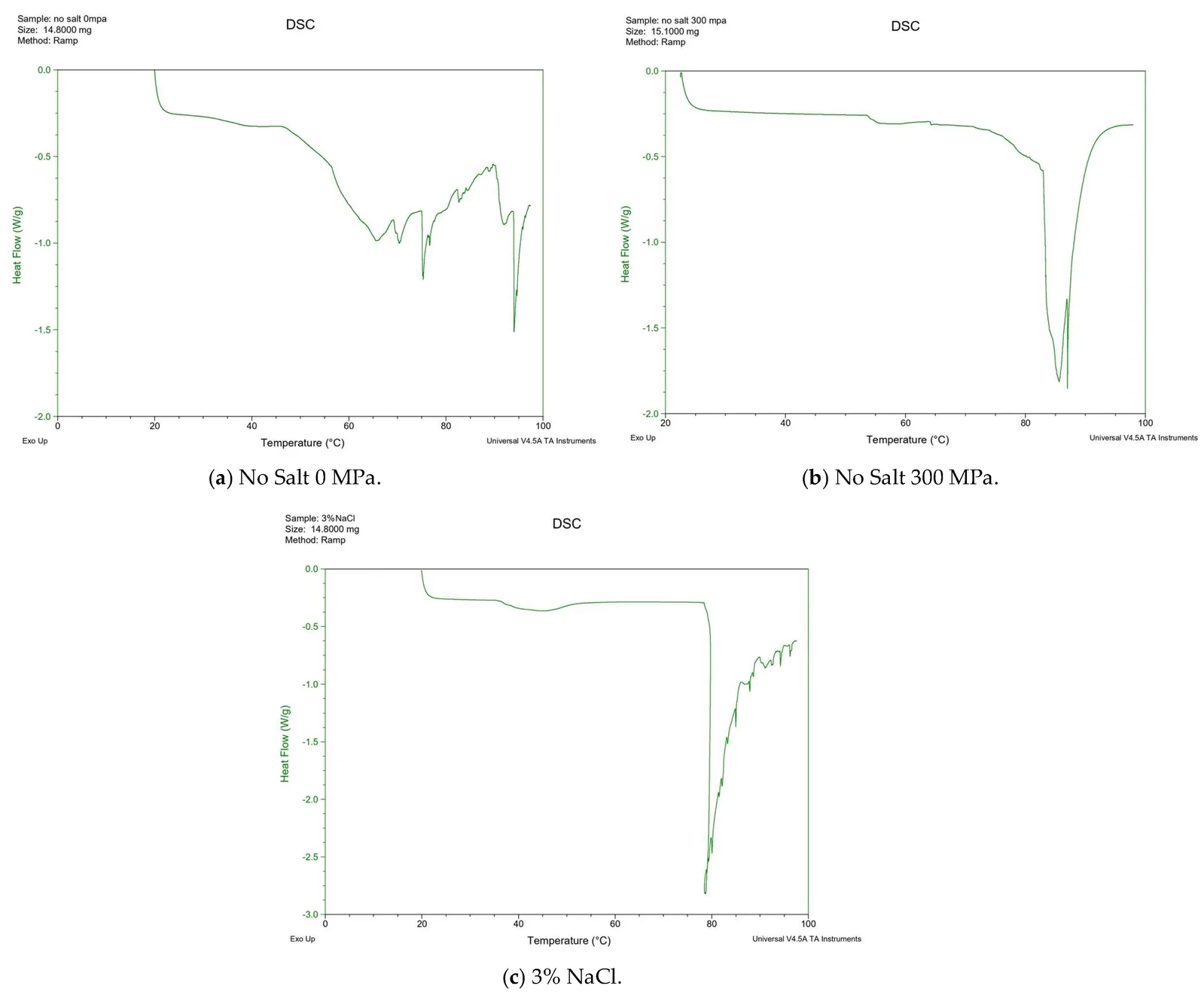
Representative DSC thermograms of hoki surimi samples showing the effects of pressure and salt on protein thermal transitions. Thermograms represent (**a**) no-salt untreated sample, 0 MPa; (**b**) no-salt pressure-treated sample, 300 MPa; and (**c**) sample prepared with 3% (*w*/*w*) NaCl relative to the fish-paste mass. Endothermic transitions indicate thermally induced protein denaturation events.

Representative DSC thermograms illustrating the effects of pressure and salt on surimi thermal behaviour are shown in Figure 4. The no-salt untreated sample, 0 MPa, exhibited weak and broad endothermic transitions across approximately 40–90 °C, indicating heterogeneous protein structures with differing thermal stability. In contrast, the no-salt pressure-treated sample, 300 MPa, showed a more defined endothermic transition centred at approximately 75–85 °C, suggesting a more uniform thermal response after pressure treatment. The salted sample, 3% NaCl relative to the fish-paste mass, exhibited a sharper dominant transition at approximately 75–80 °C with reduced lower-temperature features, indicating a more consolidated thermal profile. These differences suggest that both pressure treatment and salt addition modified the protein matrix before thermal gelation, although through different mechanisms.

These differences in peak definition and distribution suggest processing-associated modification of the protein matrix. Thermal transitions in DSC reflect the remaining thermally responsive protein fractions; therefore, changes in peak position and definition may indicate differences in protein conformation, stability, and aggregation state. The observed profiles are consistent with reported effects of salt and HPP on myofibrillar proteins, where NaCl promotes protein solubilisation and structural modification, while pressure can induce partial unfolding and aggregation [10]. In this context, the more defined profile after pressure treatment supports the interpretation that HPP altered protein organisation before heating, rather than simply changing the final gel properties after thermal processing.

Given the variability in peak clarity and the limited number of representative thermograms, DSC was interpreted descriptively rather than quantitatively. Nevertheless, the observed trends support the broader structure–function relationship identified in this study, where salt and pressure modified protein conformation in parallel with changes in water binding, cooking loss, and texture. Thus, DSC provides qualitative structural support for the interpretation that HPP influenced hoki surimi functionality through matrix-level modification of myofibrillar proteins.

### 3.7. Microstructure by Scanning Electron Microscopy (SEM)

**Figure 5:**
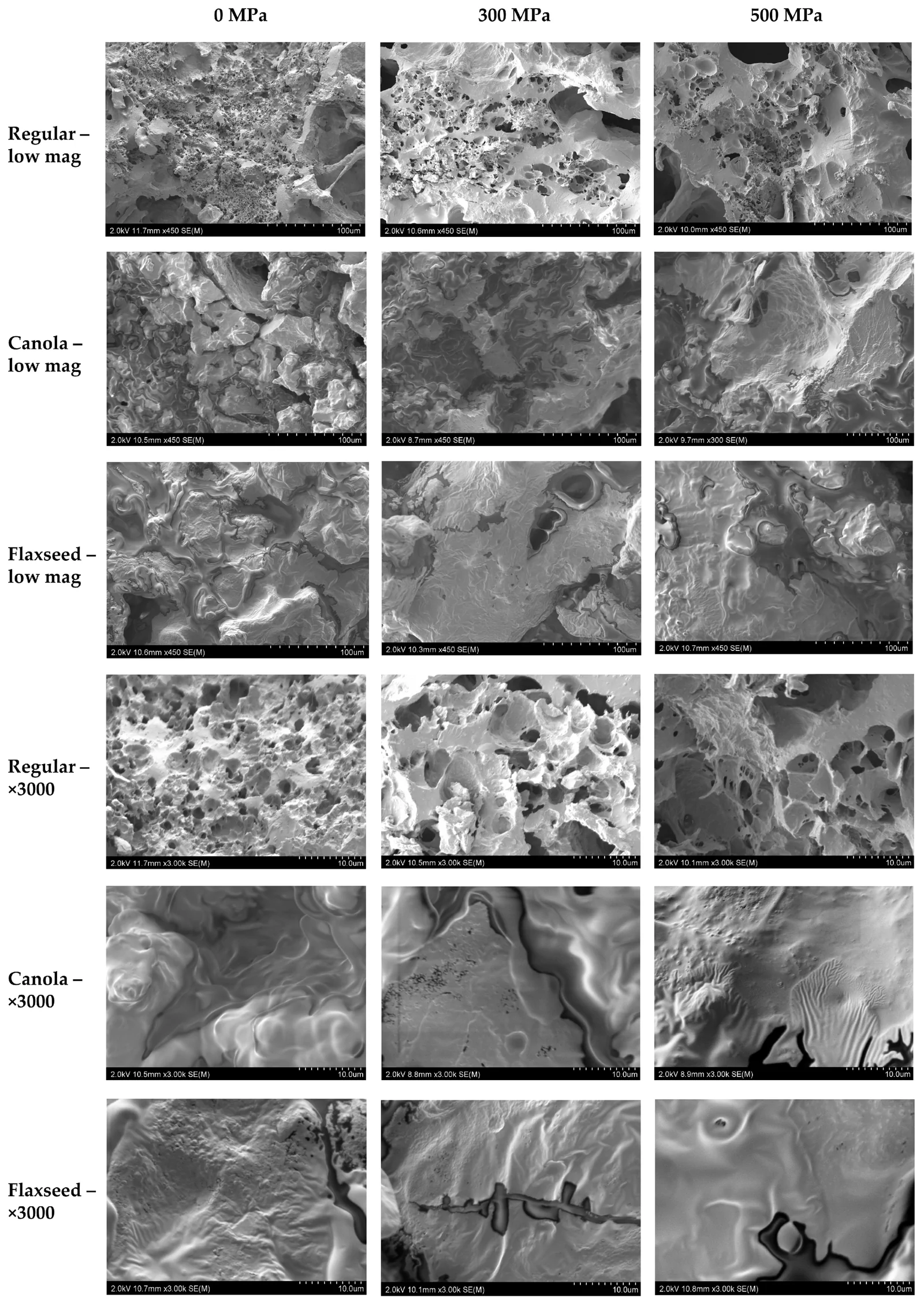
Summary of SEM observations of hoki surimi gels under different oil-fortification treatments and pressure treatments. Regular denotes the no-added-oil water control. Low-magnification images were acquired at approximately ×300–×450, and high-magnification images were acquired at ×3000.

Microstructural characteristics of surimi gels under different oil-fortification treatments and pressure treatments are summarised in Figure 5. SEM observations showed pressure-associated changes in gel network organisation. At 0 MPa, the control sample exhibited a porous and discontinuous matrix with large irregular voids, indicating weak network consolidation. With pressure treatment, the matrix became more compact and continuous, particularly at 300 MPa, where improved structural integration and more fused matrix regions were observed. This pattern is consistent with reports that moderate HPP can promote myofibrillar protein unfolding and network formation, producing denser surimi gel microstructures [10,11]. These observations provide visual support for the functional results, where pressure treatment reduced cooking loss, improved water-binding capacity, and increased slice shear force.

At 500 MPa, the matrix remained dense but appeared less uniformly organised, with local fissure-like or compact mass-like regions depending on oil-fortification treatment. This suggests that increasing pressure beyond the moderate range did not further improve network uniformity and may instead promote aggregation-dominated compaction. Similar pressure-dependent behaviour has been reported in fish protein gels, where high pressure can cause over-denaturation, aggregation, and reduced structural organisation [9,32]. Therefore, the SEM observations indicate that pressure-induced compaction and functional improvement were not equivalent. A denser matrix may improve water retention and firmness up to a point, but excessive compaction may reduce structural uniformity.

Oil incorporation modified both the baseline structure and the response to pressure. Compared with the porous control sample at 0 MPa, oil-containing samples showed distinct matrix features, including fragmented canola structures and smoother sheet-like flaxseed regions. At 300 MPa, canola samples developed a more continuous network, while flaxseed samples formed a highly fused, compact matrix. These observations indicate that lipid incorporation altered protein network organisation, although the response depended on oil-fortification treatment. Previous studies have similarly shown that lipid incorporation can modify surimi gel structure depending on lipid dispersion and compatibility with the protein matrix [6,7,8]. This suggests that the oil phase contributed to the structured gel system rather than functioning only as an added compositional component.

Overall, SEM observations support the functional results by showing that moderate pressure promoted a more integrated gel network, consistent with improved water binding, reduced cooking loss, and increased slice shear force. Higher pressure produced dense structures but did not consistently improve uniformity, indicating that pressure-induced compaction and structural organisation are not equivalent. The SEM results therefore reinforce the interpretation that surimi gel functionality reflected both pressure-induced protein restructuring and oil-fortification-dependent lipid–matrix interactions. The microstructural evidence also provides structural context for the non-linear responses observed across WBC, texture, TBARS, and FAA release: treatment effects depended not only on composition, but also on how the protein–lipid matrix was physically organised.

Frozen storage was part of the common post-processing workflow rather than an experimental factor and was therefore not evaluated separately. All samples were stored at −20 °C and thawed overnight at 4 °C, with frozen storage limited to no longer than 1 week. Nevertheless, frozen storage and thawing may have influenced water distribution, texture, oxidative behaviour, and microstructure, and this represents a limitation of the present study.

### 3.8. Protein Banding Pattern (SDS-PAGE)

**Figure 6:**
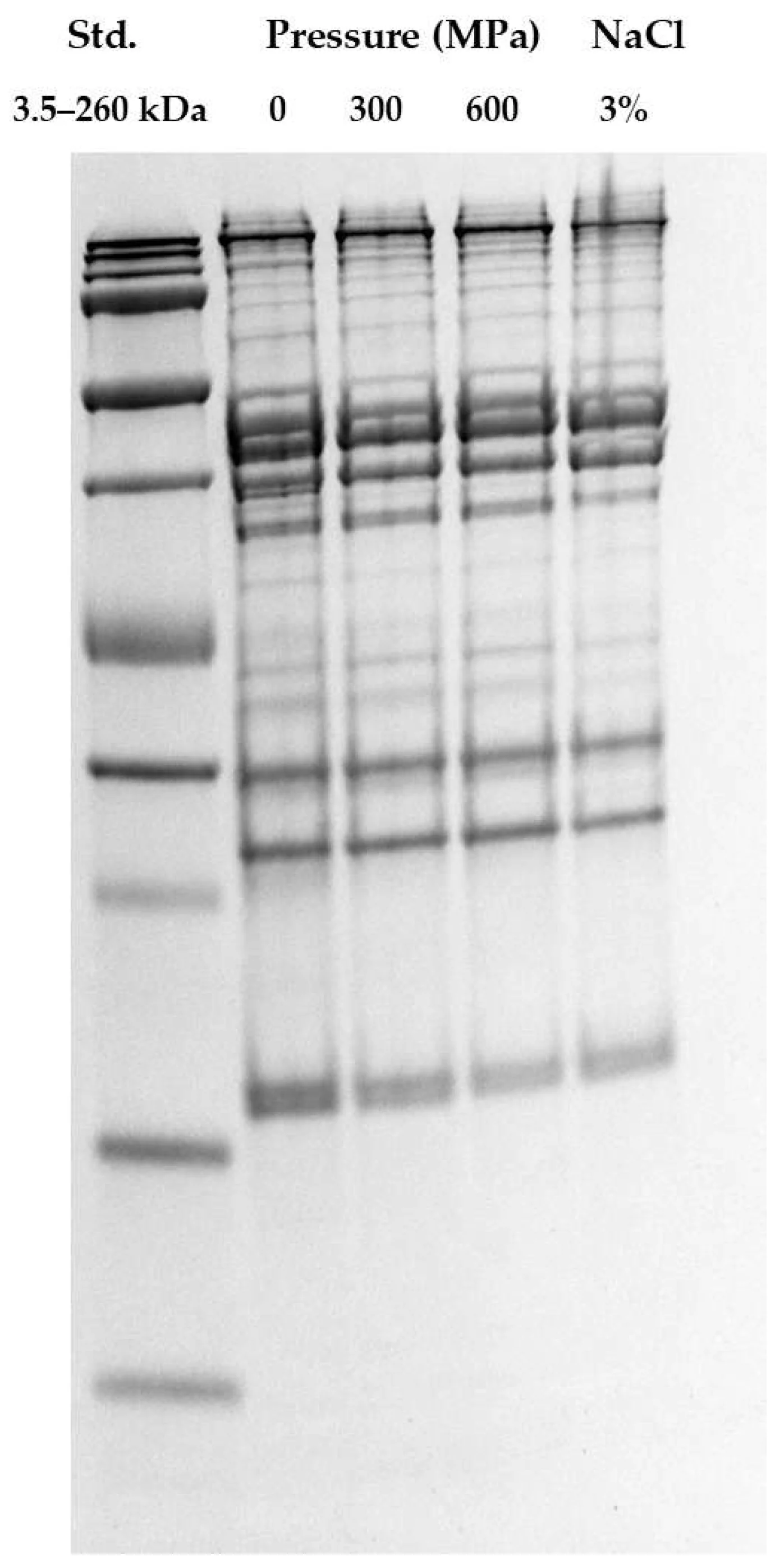
Representative SDS-PAGE profile of hoki surimi samples under different pressure and salt treatments. Lane 1: molecular-weight marker (Std.; 3.5–260 kDa); Lane 2: untreated sample, 0 MPa; Lane 3: pressure-treated sample, 300 MPa; Lane 4: pressure-treated sample, 600 MPa; Lane 5: sample prepared with 3% (*w*/*w*) NaCl relative to the fish-paste mass.

Representative SDS-PAGE profiles of surimi samples under different pressure and salt treatments are shown in Figure 6.

The SDS-PAGE profiles showed broadly similar banding patterns across untreated and pressure-treated samples, while the salted sample exhibited slight differences in band intensity and distribution. No clear pressure-dependent band shifts were observed among the 0, 300, and 600 MPa treatments. This indicates that pressure treatment did not produce major visible changes in the molecular-weight distribution of extractable proteins under the reducing conditions used.

The broadly similar profiles should not be interpreted as evidence that HPP had no structural effect. Rather, SDS-PAGE suggests that the pressure-induced changes observed in cooking loss, WBC, texture, DSC, SEM, and FAA release were not mainly associated with large-scale protein fragmentation or major electrophoretic redistribution. Under the conditions used in this study, pressure effects were more likely expressed through changes in protein conformation, aggregation state, solubility, and network organisation, which are not always clearly resolved by qualitative SDS-PAGE. This interpretation is consistent with previous reports that HPP can modify protein structure and intermolecular interactions in surimi systems without necessarily causing major electrophoretic redistribution [10].

The altered appearance of the salted sample may reflect salt-induced protein solubilisation and modification of intermolecular interactions within the surimi matrix. This supports the DSC interpretation, where the salted sample showed a more consolidated thermal profile, and is consistent with the known role of salt in modifying myofibrillar protein behaviour. Qualitative SDS-PAGE has similarly been used to assess changes in intact-protein and peptide banding patterns in protein-based in vitro digestion studies [30].

Overall, SDS-PAGE provided complementary structural evidence rather than a complete explanation of protein modification. Together with DSC and SEM, the banding profiles suggest that HPP influenced hoki surimi mainly through matrix-level restructuring instead of clear pre-digestion protein breakdown. This distinction is important for interpreting the FAA results: the marked increase in free amino acids occurred primarily during simulated intestinal digestion, indicating enzymatic hydrolysis during digestion rather than direct pressure-induced cleavage of proteins before digestion. Because SDS-PAGE was performed only under reducing conditions, disulfide-linked protein aggregation could not be assessed directly, as disulfide bonds are disrupted by the 2-mercaptoethanol-containing sample buffer. Complementary non-reducing SDS-PAGE should therefore be included in future studies to assess pressure-associated disulfide-mediated aggregation.

## 4. Conclusions

This study evaluated the combined effects of high-pressure processing (HPP) and lipid fortification on the functional, structural, oxidative, and digestion-related properties of hoki surimi. The findings show that hoki surimi behaviour reflected the combined influence of oil fortification and pressure treatment on matrix organisation, water distribution, oxidative behaviour, and enzyme accessibility within the protein–lipid gel system.

Proximate composition was influenced mainly by oil fortification, especially through changes in the moisture–fat balance, while pressure had limited effects on bulk composition. This indicates that the functional, oxidative, structural, and digestion-related responses were not explained by major compositional changes alone, but were more closely associated with matrix-level effects.

Pressure treatment improved key technological properties by reducing cooking loss and increasing water-binding capacity and slice shear force, indicating improved matrix consolidation. However, these responses were not strictly linear. Moderate pressure promoted a more integrated gel network, whereas higher pressure produced denser structures that did not consistently translate into further improvements in uniformity or functionality. SEM observations supported this interpretation by showing pressure-associated matrix consolidation alongside evidence of localised compaction at higher pressure levels. DSC and SDS-PAGE provided additional qualitative support for processing-associated structural modification, while SDS-PAGE showed no major electrophoretic redistribution of extractable protein bands.

Oil fortification influenced the surimi matrix in an oil-dependent manner. Canola and flaxseed oil treatments generally showed higher water-binding capacity than the no-added-oil control, while colour and TBARS responses reflected the optical and oxidative contributions of the incorporated oils. Flaxseed oil produced higher yellowness and TBARS values, indicating greater oxidative susceptibility, whereas canola oil showed comparatively lower oxidation. These findings identify a product-development trade-off: lipid fortification can modify functional and visual properties, but oil choice strongly affects oxidative stability.

FAA analysis showed that digestion phase was the dominant factor influencing amino acid release, with mean total FAA concentration highest after intestinal digestion. Oil fortification and pressure acted as secondary modifiers, likely through changes in matrix structure and enzyme accessibility rather than through uniform effects on protein hydrolysis. The SDS-PAGE results further suggest that the marked increase in FAAs during digestion reflected enzymatic hydrolysis rather than direct pressure-induced protein cleavage before digestion.

Overall, the results indicate that HPP and lipid fortification influenced hoki surimi quality mainly through matrix-level changes affecting water retention, texture, colour, oxidation, microstructure, and FAA release during in vitro digestion. From an application perspective, HPP may be useful for improving functional performance of hoki surimi gels, but the pressure range and oil type should be selected to balance matrix consolidation, oxidative stability, and digestion-related behaviour. Future work incorporating rheological, sensory, and swallowing-related assessments, including IDDSI-based classification where relevant, would help determine the suitability of pressure-modified surimi systems for texture-modified seafood product development.

## Figures and Tables

**Table 1:** Proximate composition of hoki surimi samples as affected by oil fortification and pressure (mean ± SD, *n* = 3). Different superscript letters within a column indicate significant differences (*p* < 0.05). Control denotes the no-added-oil water control.

Oil Fortification	Pressure (MPa)	Moisture (%)	Fat (%)	Ash (%)	Protein (%)
Control	0	72.2 ± 1.6 ^a^	1.55 ± 0.15 ^b^	3.63 ± 0.09 ^abc^	13.96 ± 0.83 ^a^
Control	200	72.7 ± 0.3 ^a^	1.70 ± 0.10 ^b^	3.54 ± 0.18 ^abc^	14.42 ± 0.24 ^a^
Control	300	72.3 ± 0.4 ^a^	1.30 ± 0.09 ^b^	3.50 ± 0.02 ^abc^	14.77 ± 0.11 ^a^
Control	400	71.9 ± 0.3 ^a^	1.34 ± 0.22 ^b^	3.43 ± 0.15 ^abc^	14.72 ± 0.12 ^a^
Control	500	73.3 ± 0.6 ^a^	1.24 ± 0.11 ^b^	3.54 ± 0.09 ^abc^	14.16 ± 0.75 ^a^
Control	600	72.3 ± 0.3 ^a^	1.29 ± 0.30 ^b^	4.11 ± 0.46 ^a^	14.39 ± 0.33 ^a^
Canola	0	66.4 ± 1.3 ^b^	8.65 ± 0.91 ^a^	3.53 ± 0.07 ^abc^	13.07 ± 0.14 ^a^
Canola	200	65.6 ± 0.1 ^b^	9.25 ± 0.30 ^a^	3.51 ± 0.26 ^abc^	14.54 ± 0.29 ^a^
Canola	300	65.3 ± 0.3 ^b^	9.11 ± 0.19 ^a^	3.82 ± 0.57 ^abc^	15.03 ± 0.44 ^a^
Canola	400	65.6 ± 0.6 ^b^	8.54 ± 0.44 ^a^	3.32 ± 0.07 ^bc^	14.26 ± 0.46 ^a^
Canola	500	65.6 ± 0.1 ^b^	8.97 ± 0.07 ^a^	3.61 ± 0.05 ^abc^	14.98 ± 2.26 ^a^
Canola	600	65.2 ± 0.5 ^b^	8.52 ± 0.32 ^a^	3.25 ± 0.34 ^bc^	14.92 ± 0.36 ^a^
Flaxseed	0	65.4 ± 1.5 ^b^	8.55 ± 0.43 ^a^	3.20 ± 0.13 ^c^	14.66 ± 0.53 ^a^
Flaxseed	200	65.1 ± 0.4 ^b^	8.65 ± 0.13 ^a^	3.91 ± 0.22 ^ab^	14.29 ± 0.23 ^a^
Flaxseed	300	65.5 ± 0.3 ^b^	9.05 ± 0.43 ^a^	3.21 ± 0.01 ^bc^	12.99 ± 0.52 ^a^
Flaxseed	400	65.6 ± 0.2 ^b^	9.21 ± 0.31 ^a^	3.26 ± 0.03 ^bc^	13.09 ± 0.69 ^a^
Flaxseed	500	64.5 ± 1.0 ^b^	9.29 ± 0.44 ^a^	3.33 ± 0.02 ^bc^	13.03 ± 1.62 ^a^
Flaxseed	600	65.9 ± 0.1 ^b^	8.85 ± 0.23 ^a^	4.13 ± 0.31 ^a^	14.72 ± 0.23 ^a^

**Table 2:** Two-way ANOVA summary showing the effects of oil fortification, pressure, and their interaction on proximate composition parameters of hoki surimi.

Parameter	Oil Fortification	Pressure	Oil × Pressure
Moisture	<0.001	0.956	0.110
Fat	<0.001	0.256	0.053
Ash	0.216	0.001	<0.001
Protein	0.026	0.314	0.012

Note: Values are *p*-values from two-factor linear models. *p* < 0.05 was considered statistically significant. Carbohydrate was excluded from statistical modelling because it was calculated by difference.

**Table 3:** Technological properties (cooking loss, WBC, and texture) of surimi samples as affected by oil fortification and pressure (mean ± SD, *n* = 3). Different superscript letters indicate significant differences among pressure levels for cooking loss and slice shear force and among oil-fortification × pressure combinations for WBC (*p* < 0.05). Control denotes the no-added-oil water control; WBC, water-binding capacity.

Oil Fortification	Pressure (MPa)	Cooking Loss (%)	WBC (%)	Texture, Slice Shear Force (N)
Control	0	7.39 ± 0.31 ^a^	66.20 ± 0.38 ^j^	64.26 ± 1.17 ^c^
Control	200	5.72 ± 0.30 ^b^	67.40 ± 0.03 ^i^	66.55 ± 1.18 ^b^
Control	300	5.63 ± 0.24 ^b^	68.18 ± 0.22 ^i^	67.44 ± 1.35 ^b^
Control	400	4.80 ± 0.14 ^c^	73.17 ± 0.46 ^g^	68.41 ± 1.37 ^a^
Control	500	5.04 ± 0.23 ^c^	74.00 ± 0.36 ^g^	69.33 ± 0.97 ^a^
Control	600	4.93 ± 0.11 ^c^	69.99 ± 0.38 ^h^	68.61 ± 1.06 ^a^
Canola	0	6.58 ± 0.06 ^a^	76.99 ± 0.36 ^d^	65.06 ± 0.93 ^c^
Canola	200	4.80 ± 0.25 ^b^	79.20 ± 0.16 ^c^	67.37 ± 1.19 ^b^
Canola	300	4.88 ± 0.18 ^b^	82.55 ± 0.31 ^ab^	68.10 ± 0.83 ^b^
Canola	400	3.70 ± 0.20 ^c^	82.98 ± 0.25 ^a^	69.08 ± 1.42 ^a^
Canola	500	3.65 ± 0.36 ^c^	83.47 ± 0.28 ^a^	70.11 ± 1.17 ^a^
Canola	600	3.91 ± 0.41 ^c^	81.84 ± 0.40 ^b^	69.23 ± 0.72 ^a^
Flaxseed	0	6.61 ± 0.29 ^a^	73.15 ± 0.02 ^g^	65.64 ± 1.18 ^c^
Flaxseed	200	4.80 ± 0.02 ^b^	74.12 ± 0.20 ^f^	67.28 ± 1.37 ^b^
Flaxseed	300	4.92 ± 0.41 ^b^	75.66 ± 0.29 ^e^	68.43 ± 0.76 ^b^
Flaxseed	400	3.46 ± 0.37 ^c^	76.23 ± 0.20 ^de^	69.31 ± 1.25 ^a^
Flaxseed	500	3.87 ± 0.30 ^c^	80.11 ± 0.31 ^c^	70.53 ± 0.97 ^a^
Flaxseed	600	4.07 ± 0.31 ^c^	76.94 ± 0.41 ^d^	69.37 ± 0.90 ^a^

**Table 4:** Two-way ANOVA summary showing the effects of oil fortification, pressure, and their interaction on technological properties of hoki surimi.

Parameter	Oil Fortification	Pressure	Oil × Pressure
Cooking loss	<0.001	<0.001	0.538
Water-binding capacity	<0.001	<0.001	<0.001
Texture, slice shear force	0.032	<0.001	1.000

Note: Values are *p*-values from two-way analysis of variance models. Values of *p* < 0.05 were considered statistically significant.

**Table 5:** Colour properties of hoki surimi samples as affected by oil fortification and pressure treatment (mean ± SD, *n* = 3). Different superscript letters within a column indicate significant differences (*p* < 0.05). Control denotes the no-added-oil water control. For b*, letters compare oil fortification × pressure combinations; for other parameters, letters compare oil-fortification treatments.

Oil Fortification	Pressure (MPa)	Lightness (L*)	Redness (a*)	Yellowness (b*)	Whiteness
Control	0	71.63 ± 0.32 ^c^	−3.31 ± 0.06 ^b^	3.94 ± 0.44 ^b^	71.17 ± 0.26 ^c^
Control	200	71.85 ± 0.46 ^c^	−3.37 ± 0.10 ^b^	3.30 ± 0.15 ^ab^	71.45 ± 0.45 ^c^
Control	300	71.98 ± 0.19 ^c^	−3.52 ± 0.20 ^b^	3.01 ± 0.25 ^a^	71.60 ± 0.19 ^c^
Control	400	71.99 ± 0.20 ^c^	−3.12 ± 0.27 ^b^	3.78 ± 0.13 ^ab^	71.56 ± 0.20 ^c^
Control	500	72.21 ± 0.27 ^c^	−3.11 ± 0.22 ^b^	3.98 ± 0.26 ^b^	71.75 ± 0.26 ^c^
Control	600	71.95 ± 0.37 ^c^	−3.15 ± 0.19 ^b^	3.02 ± 0.29 ^a^	71.61 ± 0.35 ^c^
Canola	0	76.74 ± 0.14 ^b^	−2.37 ± 0.06 ^a^	8.22 ± 0.28 ^c^	75.22 ± 0.05 ^b^
Canola	200	76.85 ± 0.42 ^b^	−2.34 ± 0.12 ^a^	8.66 ± 0.24 ^c^	75.17 ± 0.33 ^b^
Canola	300	77.30 ± 0.27 ^b^	−1.90 ± 0.26 ^a^	8.61 ± 0.34 ^c^	75.65 ± 0.15 ^b^
Canola	400	77.40 ± 0.33 ^b^	−2.22 ± 0.35 ^a^	8.58 ± 0.16 ^c^	75.72 ± 0.28 ^b^
Canola	500	77.31 ± 0.30 ^b^	−2.29 ± 0.45 ^a^	8.78 ± 0.09 ^c^	75.56 ± 0.29 ^b^
Canola	600	77.19 ± 0.29 ^b^	−2.14 ± 0.15 ^a^	8.00 ± 0.17 ^c^	75.74 ± 0.25 ^b^
Flaxseed	0	78.85 ± 0.36 ^a^	−2.43 ± 0.21 ^a^	9.59 ± 0.20 ^d^	76.64 ± 0.27 ^a^
Flaxseed	200	79.01 ± 0.26 ^a^	−2.60 ± 0.27 ^a^	10.29 ± 0.30 ^de^	76.48 ± 0.15 ^a^
Flaxseed	300	79.42 ± 0.42 ^a^	−2.45 ± 0.39 ^a^	10.39 ± 0.30 ^e^	76.81 ± 0.28 ^a^
Flaxseed	400	79.50 ± 0.18 ^a^	−2.08 ± 0.43 ^a^	9.91 ± 0.13 ^de^	77.14 ± 0.16 ^a^
Flaxseed	500	78.77 ± 0.39 ^a^	−2.57 ± 0.27 ^a^	10.15 ± 0.25 ^de^	76.32 ± 0.28 ^a^
Flaxseed	600	79.10 ± 0.16 ^a^	−2.10 ± 0.33 ^a^	10.36 ± 0.30 ^de^	76.57 ± 0.06 ^a^

**Table 6:** Two-way ANOVA summary showing the effects of oil fortification, pressure, and their interaction on colour properties of hoki surimi.

Parameter	Oil Fortification	Pressure	Oil × Pressure
L*	<0.001	0.005	0.401
a*	<0.001	0.099	0.256
b*	<0.001	0.004	<0.001
Whiteness	<0.001	0.002	0.073

Note: Values are *p*-values from two-factor linear models. *p* < 0.05 was considered statistically significant. Whiteness was calculated from L*, a*, and b* values and was analysed as a derived colour-quality index.

**Table 7:** TBARS values of hoki surimi samples as affected by oil fortification and selected pressure treatment (mean ± SD, *n* = 3). Different superscript letters indicate significant differences (*p* < 0.05). Control denotes the no-added-oil water control. TBARS, thiobarbituric acid reactive substances; MDA, malondialdehyde.

Oil Fortification	Pressure (MPa)	TBARS (mg MDA/kg Surimi)
Control	0	0.546 ± 0.008 ^g^
Control	300	0.524 ± 0.015 ^gh^
Control	500	0.506 ± 0.020 ^h^
Canola	0	1.151 ± 0.009 ^c^
Canola	300	1.075 ± 0.007 ^e^
Canola	500	1.009 ± 0.010 ^f^
Flaxseed	0	1.262 ± 0.017 ^a^
Flaxseed	300	1.184 ± 0.022 ^b^
Flaxseed	500	1.114 ± 0.008 ^d^

**Table 8:** Relative contribution of mean total free amino acid concentrations across digestion stages. Mean total FAA represents the sum of quantified individual FAAs averaged across treatments within each phase. Percentages are descriptive shares of the summed phase means rather than incremental phase-specific release. FAA, free amino acid.

Phase	Mean Total FAA (mg/kg Sample)	Relative Contribution (%)
Before	61	7.2%
Gastric	154	18.1%
Intestinal	634	74.7%

## Data Availability

The original contributions presented in this study are included in the article. Further inquiries can be directed to the corresponding author.

## Abbreviations

The following abbreviations are used in this manuscript:

ANOVA	Analysis of variance
AOAC	Association of Official Analytical Collaboration International
DSC	Differential scanning calorimetry
FAA	Free amino acid
HPLC	High-performance liquid chromatography
HPP	High-pressure processing
IDDSI	International Dysphagia Diet Standardisation Initiative
INFOGEST	Improving Health Properties of Food by Sharing Our Knowledge on the Digestive Process
LC–MS	Liquid chromatography–mass spectrometry
LC–MS/MS	Liquid chromatography–tandem mass spectrometry
MDA	Malondialdehyde
SD	Standard deviation
SEM	Scanning electron microscopy
SDS-PAGE	Sodium dodecyl sulfate–polyacrylamide gel electrophoresis
SGF	Simulated gastric fluid
SIF	Simulated intestinal fluid
SSF	Simulated salivary fluid
TBA	Thiobarbituric acid
TBARS	Thiobarbituric acid reactive substances
TCA	Trichloroacetic acid
WBC	Water-binding capacity
